# Supplementary material for: Exploring the Role of Symptom Diversity in Facial Basal Cell Carcinoma: Key Insights into Preoperative Quality of Life and Disease Progression
Source: Cancers (Basel). 2025 Jan 4;17(1):138. doi: 10.3390/cancers17010138 (PMC11720226; doi:10.3390/cancers17010138)
Supplement: Supplementary file 1 [file cancers-17-00138-s001.zip › Table S1.pdf]

Table S1. ANCOVA results for quality of life differences based on symptoms associated with facial basal cell carcinoma.

SCI Subscale	Symptom	Sum of Squares	df	Mean Square	F	p-value	Partial Eta Squared	R-squared	Adjusted R-squared
**SCI-Emotional**	Discomfort	252.03	1	252.03	6.55	**0.011**	0.0236	0.039	0.017
	Tumour	11.6	1	11.6	0.3	0.583	0.0011		
	Pain	26.44	1	26.44	0.69	0.408	0.0025		
	Itching	42.24	1	42.24	1.1	0.296	0.004		
	Erosion	24.88	1	24.88	0.65	0.422	0.0024		
	Bleeding	3.07	1	3.07	0.08	0.778	0.0003		
	Error	10425.3	271	38.47					
**SCI-Social**	Discomfort	65.3	1	65.3	5.35	**0.022**	0.0193	0.034	0.013
	Tumour	15.41	1	15.41	1.26	0.262	0.0046		

	Pain	2.18	1	2.18	0.18	0.673	0.0007		
	Itching	10.98	1	10.98	0.9	0.344	0.0033		
	Erosion	7	1	7	0.57	0.45	0.0021		
	Bleeding	0.11	1	0.11	0.01	0.924	0		
	Error	3310.77	271	12.22					
	Discomfort	37.88	1	37.88	4.06	**0.045**	0.0147		
	Tumour	15.23	1	15.23	1.63	0.203	0.006		
**SCI-Appearance**	Pain	9.39	1	9.39	1	0.317	0.0037	0.029	0.008
	Itching	1.89	1	1.89	0.2	0.653	0.0007		
	Erosion	2.12	1	2.12	0.23	0.634	0.0008		
	Bleeding	0.4	1	0.4	0.04	0.837	0.0002		

	Error	2531.12	271	9.34					
	Discomfort	906.69	1	906.69	7.69	**0.006**	0.0276		
	Tumour	126.2	1	126.2	1.07	0.302	0.0039		
	Pain	93.73	1	93.73	0.79	0.374	0.0029		
**SCI-Total**	Itching	71.22	1	71.22	0.6	0.438	0.0022	0.045	0.024
	Erosion	82.62	1	82.62	0.7	0.403	0.0026		
	Bleeding	2.13	1	2.13	0.02	0.893	0.0001		
	Error	31972.29	271	117.98					

Significance: p-value <0.05.
