# Supplementary material for: Exploring the Role of Symptom Diversity in Facial Basal Cell Carcinoma: Key Insights into Preoperative Quality of Life and Disease Progression
Source: Cancers (Basel). 2025 Jan 4;17(1):138. doi: 10.3390/cancers17010138 (PMC11720226; doi:10.3390/cancers17010138)
Supplement: Supplementary file 1 [file cancers-17-00138-s001.zip › Table S2.pdf]

Table S2. Multiple regression analysis results for quality of life subscales and associated symptoms.

SCI Subscale	Term	Coefficient	Standard Error	t-value	p-value	Confidence Interval Lower	Confidence Interval Upper	R-squared	Adjusted R-squared
**SCI-Emotional**	Intercept	26.784	3.264	8.21	1.122e-14	20.357	33.212	0.091	0.017
	Discomfort	-3.168	3.274	-0.97	0.334	-9.615	3.279		
	Tumor	2.144	3.235	0.66	0.508	-4.227	8.515		
	Pain	0.043	4.95	0.01	0.993	-9.706	9.792		
	Itching	-2.345	3.574	-0.66	0.512	-9.383	4.693		
	Erosion	0.178	3.467	0.05	0.959	-6.65	7.006		
	Bleeding	2.195	3.686	0.6	0.552	-5.063	9.454		
	Discomfort:Tumor	-0.845	3.182	-0.27	0.791	-7.111	5.422		
	Discomfort:Pain	-2.885	3.387	-0.85	0.395	-9.555	3.784		

Discomfort:Itching	-0.478	1.841	-0.26	0.795	-4.104	3.148
Discomfort:Erosion	3.984	1.838	2.17	**0.031**	0.363	7.604
Discomfort:Bleeding	0.461	1.901	0.24	0.808	-3.282	4.204
Tumor:Pain	-2.057	4.75	-0.43	0.665	-11.411	7.297
Tumor:Itching	2.131	3.135	0.68	0.497	-4.043	8.305
Tumor:Erosion	-1.117	3.252	-0.34	0.732	-7.522	5.288
Tumor:Bleeding	-2.407	3.135	-0.77	0.443	-8.581	3.767
Pain:Itching	6.745	3.49	1.93	0.054	-0.129	13.618
Pain:Erosion	3.689	5.347	0.69	0.491	-6.841	14.22
Pain:Bleeding	-6.491	5.443	-1.19	0.234	-17.21	4.229
Itching:Erosion	-1.269	1.951	-0.65	0.516	-5.11	2.572

	Itching:Bleeding	1.207	1.925	0.63	0.531	-2.583	4.997		
	Erosion:Bleeding	-0.867	2.103	-0.41	0.68	-5.009	3.275		
**SCI-Social**	Intercept	22.515	1.862	12.09	6.264e-27	18.849	26.182	0.065	-0.012
	Discomfort	-1.67	1.868	-0.89	0.372	-5.348	2.007		
	Tumor	0.321	1.846	0.17	0.862	-3.314	3.955		
	Pain	0.073	2.824	0.03	0.979	-5.488	5.634		
	Itching	-2.2	2.039	-1.08	0.282	-6.215	1.815		
	Erosion	-0.168	1.978	-0.08	0.932	-4.063	3.727		
	Bleeding	-0.068	2.103	-0.03	0.974	-4.208	4.073		
	Discomfort:Tumor	0.045	1.815	0.02	0.98	-3.53	3.619		
	Discomfort:Pain	0.982	1.932	0.51	0.612	-2.823	4.787		

Discomfort:Itching	0.224	1.05	0.21	0.831	-1.844	2.293
Discomfort:Erosion	1.79	1.049	1.71	0.089	-0.276	3.855
Discomfort:Bleeding	-1.323	1.084	-1.22	0.224	-3.458	0.813
Tumor:Pain	-1.85	2.71	-0.68	0.495	-7.186	3.486
Tumor:Itching	2.24	1.788	1.25	0.212	-1.282	5.762
Tumor:Erosion	-0.522	1.855	-0.28	0.779	-4.175	3.132
Tumor:Bleeding	0.446	1.788	0.25	0.803	-3.076	3.968
Pain:Itching	1.021	1.991	0.51	0.608	-2.9	4.943
Pain:Erosion	0.147	3.05	0.05	0.962	-5.861	6.154
Pain:Bleeding	-0.946	3.105	-0.3	0.761	-7.061	5.169
Itching:Erosion	-0.552	1.113	-0.5	0.62	-2.743	1.639

	Itching:Bleeding	-0.112	1.098	-0.1	0.919	-2.274	2.05		
	Erosion:Bleeding	0.742	1.2	0.62	0.537	-1.62	3.105		
**SCI-Appearance**	Intercept	13.07	1.621	8.06	2.884e-14	9.878	16.263	0.068	-0.009
	Discomfort	-1.682	1.626	-1.03	0.302	-4.885	1.52		
	Tumor	-0.019	1.607	-0.01	0.991	-3.183	3.146		
	Pain	-1.847	2.459	-0.75	0.453	-6.689	2.995		
	Itching	0.162	1.775	0.09	0.927	-3.334	3.658		
	Erosion	-0.387	1.722	-0.22	0.822	-3.779	3.004		
	Bleeding	-0.289	1.831	-0.16	0.875	-3.894	3.316		
	Discomfort:Tumor	0.289	1.58	0.18	0.855	-2.824	3.401		
	Discomfort:Pain	-0.989	1.682	-0.59	0.557	-4.302	2.324		

Discomfort:Itching	-0.64	0.915	-0.7	0.485	-2.441	1.161
Discomfort:Erosion	1.793	0.913	1.96	0.051	-0.005	3.592
Discomfort:Bleeding	-0.387	0.944	-0.41	0.682	-2.246	1.472
Tumor:Pain	1.964	2.359	0.83	0.406	-2.683	6.61
Tumor:Itching	0.227	1.557	0.15	0.884	-2.839	3.294
Tumor:Erosion	-0.446	1.616	-0.28	0.783	-3.627	2.736
Tumor:Bleeding	0.886	1.557	0.57	0.57	-2.181	3.953
Pain:Itching	2.883	1.734	1.66	0.098	-0.531	6.297
Pain:Erosion	0.779	2.656	0.29	0.769	-4.451	6.01
Pain:Bleeding	-3.449	2.704	-1.28	0.203	-8.774	1.875
Itching:Erosion	0.475	0.969	0.49	0.624	-1.433	2.383

	Itching:Bleeding	-0.547	0.956	-0.57	0.567	-2.43	1.335		
	Erosion:Bleeding	-0.311	1.045	-0.3	0.767	-2.368	1.747		
**SCI-Total**	Intercept	49.299	4.688	10.52	9.856e-22	40.067	58.531	0.085	0.01
	Discomfort	-4.839	4.702	-1.03	0.304	-14.098	4.421		
	Tumor	2.465	4.647	0.53	0.596	-6.686	11.616		
	Pain	0.116	7.11	0.02	0.987	-13.886	14.118		
	Itching	-4.545	5.133	-0.89	0.377	-14.654	5.564		
	Erosion	0.01	4.98	0	0.998	-9.797	9.817		
	Bleeding	2.128	5.294	0.4	0.688	-8.297	12.553		
	Discomfort:Tumor	-0.8	4.57	-0.18	0.861	-9.8	8.2		
	Discomfort:Pain	-1.903	4.865	-0.39	0.696	-11.483	7.677		

Discomfort:Itching	-0.254	2.645	-0.1	0.924	-5.462	4.955
Discomfort:Erosion	5.774	2.641	2.19	**0.03**	0.573	10.974
Discomfort:Bleeding	-0.861	2.73	-0.32	0.753	-6.238	4.515
Tumor:Pain	-3.906	6.822	-0.57	0.567	-17.342	9.529
Tumor:Itching	4.371	4.503	0.97	0.333	-4.497	13.238
Tumor:Erosion	-1.639	4.672	-0.35	0.726	-10.838	7.561
Tumor:Bleeding	-1.961	4.503	-0.44	0.664	-10.829	6.906
Pain:Itching	7.766	5.013	1.55	0.123	-2.106	17.639
Pain:Erosion	3.836	7.681	0.5	0.618	-11.289	18.961
Pain:Bleeding	-7.437	7.819	-0.95	0.342	-22.834	7.96
Itching:Erosion	-1.821	2.802	-0.65	0.516	-7.339	3.696

Itching:Bleeding	1.095	2.764	0.4	0.692	-4.349	6.539
Erosion:Bleeding	-0.125	3.021	-0.04	0.967	-6.074	5.824

Significance: p-value <0.05.
