# Supplementary material for: Exploring the Role of Symptom Diversity in Facial Basal Cell Carcinoma: Key Insights into Preoperative Quality of Life and Disease Progression
Source: Cancers (Basel). 2025 Jan 4;17(1):138. doi: 10.3390/cancers17010138 (PMC11720226; doi:10.3390/cancers17010138)
Supplement: Supplementary file 1 [file cancers-17-00138-s001.zip › Table S3.pdf]

Table S3. Multiple Regression analyses for symptoms and histological tumor types.

Histological Type	Variable	Coefficient	Standard Error	P-Value	95% CI Lower	95% CI Upper	McFadden's R^2	AIC	LR Test P-Value
**Nodular BCC**	const	1.092194	0.548102	0.046296	0.017935	2.166453	0.011475	287.3786	0.786785
	Discomfort	0.209801	0.309602	0.497996	-0.39701	0.81661			
	Tumor	0.233598	0.500024	0.640375	-0.74643	1.213628			
	Pain	-0.21185	0.543008	0.69643	-1.27613	0.852425			
	Itching	0.105408	0.33431	0.752532	-0.54983	0.760643			
	Erosion	0.378166	0.364694	0.299763	-0.33662	1.092953			
	Bleeding	-0.56509	0.365601	0.122187	-1.28166	0.15147			
**Superficial BCC**	const	-0.98414	0.604019	0.103246	-2.16799	0.199717	0.04064	233.7378	0.156964
	Discomfort	-0.04651	0.358969	0.896907	-0.75008	0.657055			
	Tumor	-0.50178	0.549168	0.360866	-1.57813	0.574565			
	Pain	-1.36198	1.050127	0.194644	-3.42019	0.696236			
	Itching	0.177862	0.380705	0.640363	-0.56831	0.92403			
	Erosion	0.015811	0.38613	0.967339	-0.74099	0.772611			
	Bleeding	-0.92514	0.433711	0.032918	-1.7752	-0.07508			
**Infiltrative BCC**	const	-0.28954	0.480443	0.546743	-1.23119	0.652113	0.049243	342.5063	0.00923
	Discomfort	0.310173	0.277266	0.263275	-0.23326	0.853604			
	Tumor	-1.21202	0.431095	0.004931	-2.05695	-0.36709			
	Pain	0.071303	0.491149	0.884572	-0.89133	1.033938			
	Itching	-0.17248	0.291004	0.553377	-0.74284	0.397877			
	Erosion	0.588841	0.321074	0.066658	-0.04045	1.218136			
	Bleeding	0.200489	0.309046	0.51651	-0.40523	0.806209			

Significance: p-value <0.05.
