# Supplementary material for: Exploring the Role of Symptom Diversity in Facial Basal Cell Carcinoma: Key Insights into Preoperative Quality of Life and Disease Progression
Source: Cancers (Basel). 2025 Jan 4;17(1):138. doi: 10.3390/cancers17010138 (PMC11720226; doi:10.3390/cancers17010138)
Supplement: Supplementary file 1 [file cancers-17-00138-s001.zip › Table S4.pdf]

Table S4. Multiple logistic regression analysis for presence of symptoms and care-seeking behavior within 6 months.

Variable	Coef	Std Err	z	P>|z|	[0.025	0.975]
const	1.1606	1.363	0.851	0.395	-1.511	3.832
Discomfort	-0.1492	1.354	-0.11	0.912	-2.803	2.504
Anxiety	0.0763	1.25	0.061	0.951	-2.374	2.527
Tumor	-1.9369	1.348	-1.437	0.151	-4.579	0.705
Pain	-3.1268	2.223	-1.406	0.16	-7.484	1.231
Itching	-3.0062	1.717	-1.751	0.08	-6.371	0.359
Erosion	-1.3986	1.399	-1	0.318	-4.141	1.344
Bleeding	-1.0791	1.46	-0.739	0.46	-3.941	1.782
Discomfort_x_Anxiety	-0.186	0.622	-0.299	0.765	-1.406	1.034
Discomfort_x_Tumor	0.2066	1.316	0.157	0.875	-2.373	2.786
Discomfort_x_Pain	1.4057	1.503	0.935	0.35	-1.54	4.351
Discomfort_x_Itching	0.4703	0.706	0.666	0.505	-0.913	1.854
Discomfort_x_Erosion	-0.5584	0.718	-0.778	0.436	-1.965	0.848
Discomfort_x_Bleeding	0.2212	0.752	0.294	0.769	-1.253	1.696

Anxiety_x_Tumor	-0.227	1.189	-0.191	0.849	-2.558	2.104
Anxiety_x_Pain	2.1619	1.418	1.524	0.127	-0.618	4.942
Anxiety_x_Itching	0.5345	0.655	0.816	0.415	-0.749	1.818
Anxiety_x_Erosion	-0.4412	0.723	-0.61	0.542	-1.859	0.976
Anxiety_x_Bleeding	0.5723	0.741	0.772	0.44	-0.88	2.025
Tumor_x_Pain	1.3612	1.746	0.78	0.436	-2.061	4.784
Tumor_x_Itching	2.576	1.5	1.717	0.086	-0.364	5.517
Tumor_x_Erosion	1.3387	1.358	0.986	0.324	-1.323	4.001
Tumor_x_Bleeding	0.1959	1.348	0.145	0.884	-2.446	2.838
Pain_x_Itching	-1.9248	1.371	-1.404	0.16	-4.611	0.762
Pain_x_Erosion	2.1772	2.281	0.955	0.34	-2.293	6.647
Pain_x_Bleeding	-0.1405	2.395	-0.059	0.953	-4.835	4.554
Itching_x_Erosion	0.0263	0.772	0.034	0.973	-1.487	1.54
Itching_x_Bleeding	0.3597	0.765	0.47	0.638	-1.139	1.858
Erosion_x_Bleeding	0.6319	0.849	0.744	0.457	-1.032	2.296
