# Supplementary material for: Exploring the Role of Symptom Diversity in Facial Basal Cell Carcinoma: Key Insights into Preoperative Quality of Life and Disease Progression
Source: Cancers (Basel). 2025 Jan 4;17(1):138. doi: 10.3390/cancers17010138 (PMC11720226; doi:10.3390/cancers17010138)
Supplement: Supplementary file 1 [file cancers-17-00138-s001.zip › Table S5.pdf]

Table S5. Multiple logistic regression analysis for presence of symptoms and care-seeking behavior within 12 months.

Variable	Coefficient	Standard Error	z-Value	p-Value	95% CI (Lower)	95% CI (Upper)
Tumor	-5.3836	2.617	-2.057	0.04	-10.513	-0.254
Pain	-6.3793	2.958	-2.157	0.031	-12.177	-0.582
Itching	-2.8189	1.721	-1.638	0.101	-6.191	0.553
Discomfort	-3.1568	1.748	-1.806	0.071	-6.582	0.268
Anxiety	0.0128	1.509	0.009	0.993	-2.944	2.969
Erosion	-1.3426	1.667	-0.805	0.421	-4.611	1.925
Bleeding	-3.3872	2.051	-1.652	0.099	-7.407	0.632
Tumor x Itching	3.5571	1.558	2.283	0.022	0.503	6.611
Discomfort x Tumor	3.1494	1.676	1.879	0.06	-0.136	6.435
Discomfort x Anxiety	-0.497	0.574	-0.865	0.387	-1.623	0.629
Discomfort x Pain	1.8893	1.69	1.118	0.264	-1.424	5.202
Discomfort x Itching	-0.2253	0.66	-0.341	0.733	-1.519	1.068
Discomfort x Erosion	-0.1155	0.643	-0.18	0.857	-1.376	1.145
Discomfort x Bleeding	1.0201	0.668	1.526	0.127	-0.29	2.33

Anxiety x Tumor	0.1528	1.42	0.108	0.914	-2.63	2.935
Anxiety x Pain	1.9078	1.5	1.272	0.203	-1.031	4.847
Anxiety x Itching	0.6326	0.612	1.033	0.302	-0.568	1.833
Anxiety x Erosion	-0.2424	0.639	-0.379	0.705	-1.495	1.011
Anxiety x Bleeding	1.1886	0.639	1.861	0.063	-0.064	2.441
Tumor x Pain	2.7194	2.407	1.13	0.259	-1.999	7.438
Tumor x Erosion	1.1434	1.57	0.728	0.467	-1.935	4.222
Tumor x Bleeding	1.3326	1.926	0.692	0.489	-2.443	5.108
Pain x Itching	-1.8069	1.396	-1.295	0.195	-4.543	0.929
Pain x Erosion	2.7745	2.639	1.051	0.293	-2.398	7.947
Pain x Bleeding	0.1276	2.837	0.045	0.964	-5.433	5.688
Itching x Erosion	-0.8355	0.703	-1.188	0.235	-2.213	0.543
Itching x Bleeding	-0.1197	0.676	-0.177	0.859	-1.445	1.205
Erosion x Bleeding	0.6549	0.77	0.851	0.395	-0.854	2.164
